# CCMetagen: comprehensive and accurate identification of eukaryotes and prokaryotes in metagenomic data

**DOI:** 10.1186/s13059-020-02014-2

**Published:** 2020-04-28

**Authors:** Vanessa R. Marcelino, Philip T. L. C. Clausen, Jan P. Buchmann, Michelle Wille, Jonathan R. Iredell, Wieland Meyer, Ole Lund, Tania C. Sorrell, Edward C. Holmes

**Affiliations:** 1 Marie Bashir Institute for Infectious Diseases and Biosecurity and Faculty of Medicine and Health, Sydney Medical School, Westmead Clinical School, The University of Sydney, Sydney, NSW 2006 Australia; 2 Centre for Infectious Diseases and Microbiology, Westmead Institute for Medical Research, Westmead, NSW 2145 Australia; 3 School of Life & Environmental Sciences, Charles Perkins Centre, The University of Sydney, Sydney, NSW 2006 Australia; 4 National Food Institute, Technical University of Denmark, 2800 Kgs Lyngby, Denmark; 5 WHO Collaborating Centre for Reference and Research on Influenza, The Peter Doherty Institute for Infection and Immunity, Melbourne, VIC 3000 Australia; 6 Westmead Hospital (Research and Education Network), Westmead, NSW 2145 Australia; 7 Molecular Mycology Research Laboratory, Centre for Infectious Diseases and Microbiology, Westmead Institute for Medical Research, Westmead, NSW 2145 Australia

**Keywords:** Microbiome, Metagenomic classifier, ConClave sorting, Fungi

## Abstract

There is an increasing demand for accurate and fast metagenome classifiers that can not only identify bacteria, but all members of a microbial community. We used a recently developed concept in read mapping to develop a highly accurate metagenomic classification pipeline named CCMetagen. The pipeline substantially outperforms other commonly used software in identifying bacteria and fungi and can efficiently use the entire NCBI nucleotide collection as a reference to detect species with incomplete genome data from all biological kingdoms. CCMetagen is user-friendly, and the results can be easily integrated into microbial community analysis software for streamlined and automated microbiome studies.

## Background

Microbial communities in natural and host-associated environments commonly harbor a mix of bacteria, archaea, viruses, and microbial eukaryotes. Bacterial diversity has been extensively studied with high-throughput sequencing (HTS) targeting 16S rDNA markers [1, 2]. However, these do not amplify eukaryotic sequences, and our knowledge on the diversity and distribution of microbial eukaryotes is limited [3, 4]. Although there is an increasing number of studies using eukaryotic-specific markers, these are relatively uncommon and face multiple methodological limitations [5, 6]. The problematic amplification step can be bypassed by sequencing the total DNA (metagenome) or RNA (metatranscriptome) in a sample to characterize all the genes contained or expressed within it. Metagenomics and metatranscriptomics are promising tools to bridge the knowledge gap in the diversity of microbial eukaryotes because they are essentially kingdom-agnostic, are less susceptible to amplification bias, and yield a large set of genes that can be used for taxonomic identification.

Multiple software packages have been developed to reveal the species composition of metagenomic samples (reviewed in [7]). While well-known bacterial species can be easily identified at the species and strain levels [8, 9], it remains challenging to obtain a fine-grained taxonomic classification of lesser-known species and microbial eukaryotes [10, 11]. Many of the current metagenomic classifiers assign a taxonomy to individual short sequence reads [7]. However, as closely related species share very similar or identical genome segments, short reads often map to multiple species in the reference data set. Some metagenomic classifiers, like MEGAN [12] and Kraken [13], address this issue by calculating the lowest common ancestor (LCA) among all species sharing those sequences. Paradoxically, as identical regions in reference databases become more common, fewer reads can be classified at the species level [14]. Other classifiers use a database of clade-specific diagnostic regions (e.g., [9]). While highly accurate, this procedure relies heavily on reference databases of complete genomes, which often cannot be readily updated by the end user. Complete genomes are available for only a small fraction of the microbial eukaryotic species. For example, as of April 2019, the widely used NCBI RefSeq database contained 285 fungal genome sequences, even though it is estimated that there are over 2 million species of fungi [15]. Therefore, relying on these databases of complete genomes greatly restricts the inclusion of microbial eukaryotes in metagenome studies.

**Fig. 1:**
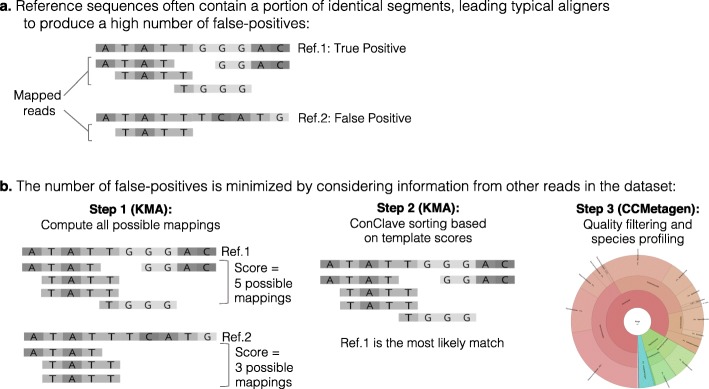
Overview of the ConClave sorting scheme applied to species identification in metagenomic data sets. The figure represents a data set containing 5 sequence reads (4 bp) and two closely related reference sequences (templates), including a true positive (Ref. 1) and a potential false positive (Ref. 2). **a** Commonly used read mappers yield a high number of false positives because reads can be randomly assigned to closely related reference sequences sharing identical fragments spanning the whole sequence read (represented by the ATATT region). **b** The KMA aligner minimizes this problem by scoring reference sequences based on all possible mappings of all reads and then choosing the templates with the highest scores. Coupled with KMA, CCMetagen produces highly accurate taxonomic assignments of reads in metagenomic data sets in user-friendly formats

A recently developed concept in read mapping—the ConClave sorting scheme, implemented in the KMA software [16]—is more accurate than other mapping strategies as it takes advantage of the information from all reads in the data set (Fig. 1). Our goal was to use this approach to produce an accurate metagenomic classification pipeline that will allow the inclusion of microbial eukaryotes in metagenomic studies. We now present a novel tool—CCMetagen (ConClave-based Metagenomics)—to process KMA sequence alignments and produce accurate taxonomic classifications from metagenomic data. We benchmark CCMetagen using simulated fungal and bacterial metagenomes and metatranscriptomes. Additionally, we include two case studies with real biological data to demonstrate that CCMetagen effectively produces a comprehensive overview of the eukaryotic and prokaryotic members of microbial communities.

## Results

### Implementation and availability

Metagenomic reads (or contigs) are first mapped against a reference database with KMA [16], which implements the ConClave sorting scheme for better-informed and highly accurate alignments (Fig. 1). CCMetagen is then used to perform quality filtering and produce taxonomic classifications that can be explored in text or interactive visualization formats (Krona plots [17]). Our pipeline uses the NCBI taxonomic database (taxids) to produce ranked and updated taxonomic classifications, so that the ever-changing species nomenclature issue is minimized [18]. CCMetagen yields classifications at a taxonomic level that reflects the similarity between the query and reference sequences. This ranked classification means that species with only distant relatives in reference databases (e.g., undescribed genera) can be identified, as well as well-known microorganisms. The output of CCMetagen can be easily converted into a PhyloSeq object for statistical analyses in R [19]. The pipeline is sufficiently fast to use the entire NCBI nucleotide collection (nt) as a reference database [20], thereby enabling the inclusion of microbial eukaryotes—in addition to bacteria, viruses, and archaea—in metagenome surveys. Our program is implemented in Python 3 and is freely available at https://github.com/vrmarcelino/CCMetagen [21] or via the Python Package Index (PyPi) [22]. A web service to easily run the pipeline with default settings is available at https://cge.cbs.dtu.dk/services/ccmetagen/ [23].

### Fungal classifications are more accurate with the CCMetagen pipeline

To test the performance of CCMetagen in identifying an important and diverse group of microbial eukaryotes, we simulated in silico a fungal metatranscriptome (15 species) and a fungal metagenome (30 species). We then benchmarked CCMetagen’s performance by comparing it with widely used metagenomic classification software, including Centrifuge [24], Kraken2 [25], and KrakenUniq [26]. These programs were chosen because they are compatible with custom-made reference databases, which is a desirable flexibility when working with microbial eukaryotes. KrakenUniq was recently shown to outperform eleven other classification methods when using the NCBI nucleotide collection (“nt” database), including Diamond/Blast + MEGAN [12, 27, 28], CLARK [29], GOTTCHA [30], PhyloSift [31], and MetaPhlAn2 [9]. KrakenUniq therefore provides a gold standard for the available tools. We evaluated precision, recall, and F1 scores of the benchmarked software in identifying fungal taxa in the simulated fungal metagenome and metatranscriptome (see the “Methods” section). The F1 score is the harmonic average of precision and recall; high F1 scores can be interpreted as a good trade-off between precision and recall.

**Fig. 2:**
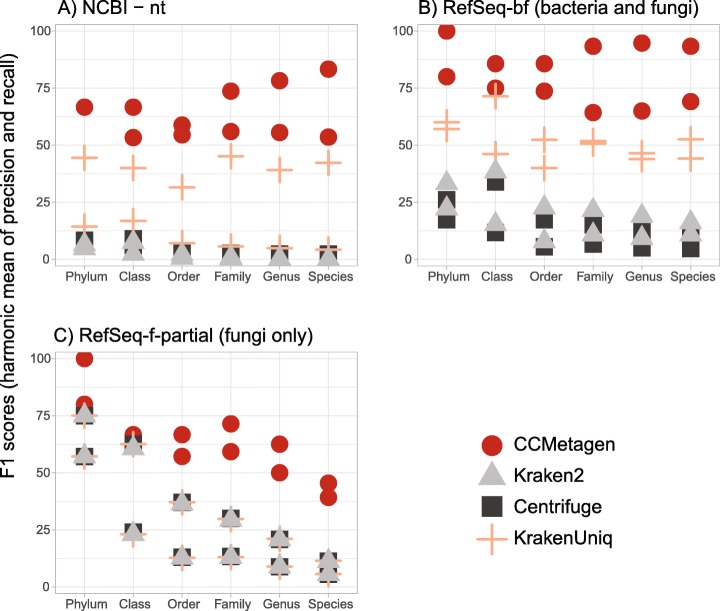
The CCMetagen pipeline has a higher F1 score than other metagenomic classification methods for all taxonomic ranks. The two points for each program and taxonomic rank represent the results using a simulated metagenome and a metatranscriptome sample of a fungal community. **a** Results using the whole NCBI nt collection as a reference database. **b** Results using the RefSeq-bf (bacteria and fungi) database, containing all bacterial and fungal genomes available. **c** Partial RefSeq database containing only some of the fungal species currently present in the RefSeq-bf database, mimicking the effects of dealing with species without representatives in reference data sets. In this case, Kraken2, Centrifuge, and KrakenUniq have overlapping results. Refer to Additional file 1: Figures S1 and S2 and Additional file 2 for more information, including precision and recall

The CCMetagen pipeline achieved the highest precision and F1 scores of all the approaches tested (Fig. 2, Additional file 1: Figures S1 and S2, Additional file 2). KrakenUniq achieved higher precision than Kraken2 and Centrifuge when using an ideal database (i.e., RefSeq-bf, which contains only the complete and curated genomes of fungi and bacteria, containing all species from the test data set). However, the performance of KrakenUniq decreased substantially when the database was incomplete (i.e., RefSeq-f-partial, where a part of the reference sequences was removed to mimic the effects of handling species without reference genomes).

Centrifuge, Kraken2, and KrakenUniq yielded many more taxa than the number included in the test data sets: for example, Centrifuge, when used with the nt database, reported 6950 species in the simulated metagenome containing 30 species, while CCMetagen yielded only 15. Naturally, their recall was very high—Centrifuge and KrakenUniq recovered 100% of the taxa present in the test data set when using the RefSeq-bf and nt reference databases (Additional file 1: Figure S2). The species-level recall of Kraken2 decreased when using the nt database. CCMetagen recovered between 50 and 100% of the species when used with RefSeq-bf and nt databases (Additional file 2).

We also tested CCMetagen with assembled sequence reads (Additional file 3). When using the NCBI nt collection, precision ranged from 67 to 71% for species-level classifications, while recall ranged from 53 to 100% (Additional file 4), indicating that our pipeline is suited to processing long sequences.

The fastest processing time was achieved by Kraken2 (Table 1). The combined CPU time of KMA and CCMetagen (i.e., the CCMetagen pipeline) was faster than Centrifuge and KrakenUniq when using the whole NCBI nt database, but it was the slowest approach when using the RefSeq database. The KMA indexing of the nt database was limited to only include *k-*mers with a two-letter prefix, which on average corresponds to only saving non-overlapping *k-*mers. This prefixing substantially increases the speed and could also be applied to the RefSeq database if a faster processing time is required (Additional file 3). Choosing a longer prefix will result in gaps in the database which in turn will result in lower precision and recall. With a prefix of two, this is relatively limited. When the NCBI nt data set was used, CCMetagen required ~ 15 min to process a sample (~ 5 Gb, 7.8M reads on average).

**Table 1:** CPU time (in minutes) required to analyze a simulated fungal metatranscriptome (mtt, ~ 9M PE reads) and a fungal metagenome (mtg, ~ 6.7M PE reads)

	nt		RefSeq-bf		RefSeq-f-Partial	
	mtt	mtg	mtt	mtg	mtt	mtg
Kraken2	10.92	7.05	5.29	3.98	4.48	3.50
CCMetagen*	17.24	13.54	85.74	67.00	69.29	20.58
Centrifuge	40.11	27.54	23.70	19.41	16.67	16.10
KrakenUniq	74.11	74.94	43.33	40.85	29.65	21.04

*The CCMetagen time was calculated as the sum of the CPU time used by KMA and CCMetagen

### Bacterial communities are best depicted with the CCMetagen pipeline

**Fig. 3:**
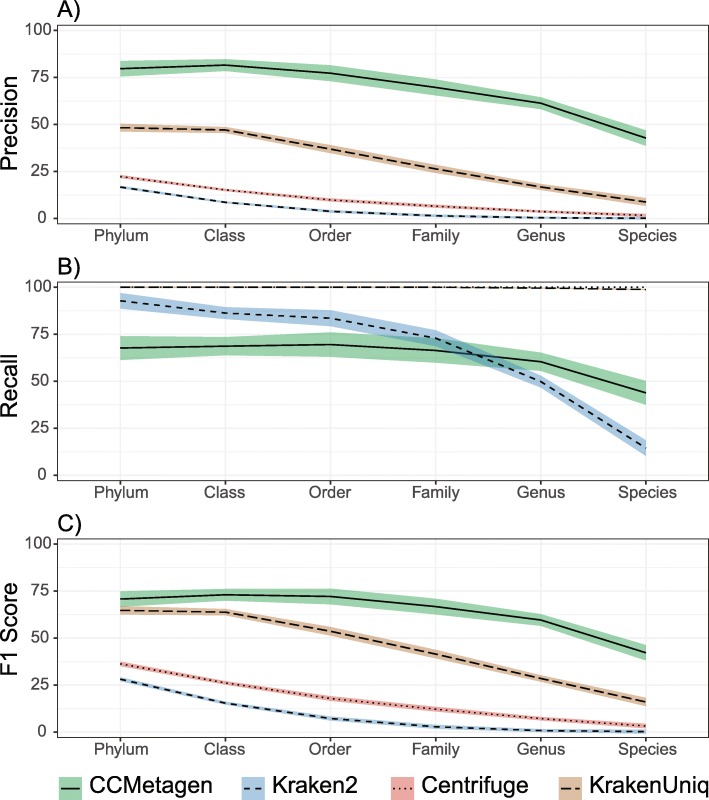
CCMetagen pipeline performance for bacterial classifications, compared with Kraken2, Centrifuge, and KrakenUniq. Precision (% of true positives), recall (% of taxa identified), and F1 scores represent averages across 10 simulated metagenome samples. Shaded areas indicate 75% confidence intervals

We assessed the performance of the CCMetagen pipeline when applied to 10 bacterial communities simulated at different levels of complexity [32, 33]. Using the NCBI nt collection as a reference, CCMetagen achieved the highest precision and F1 scores at all taxonomic ranks (Fig. 3). Recall was highest for Centrifuge and KrakenUniq. In this data set, the recall of Kraken2 was higher than CCMetagen from phylum- to family-level classifications, but lower than CCMetagen at the genus and species level.

The complete CCMetagen pipeline (KMA + CCMetagen) required an average of 2.1 min to process the bacterial metagenomes (± 0.26 SD). It was slower than Kraken2 (average 0.27 m, ± 0.21 SD) and faster than KrakenUniq (average 2.56 m, ± 2.60 SD) and Centrifuge (average 9.19 m, ± 0.80 SD).

### Biological data set 1: Experimentally seeded fungal metatranscriptome

**Fig. 4:**
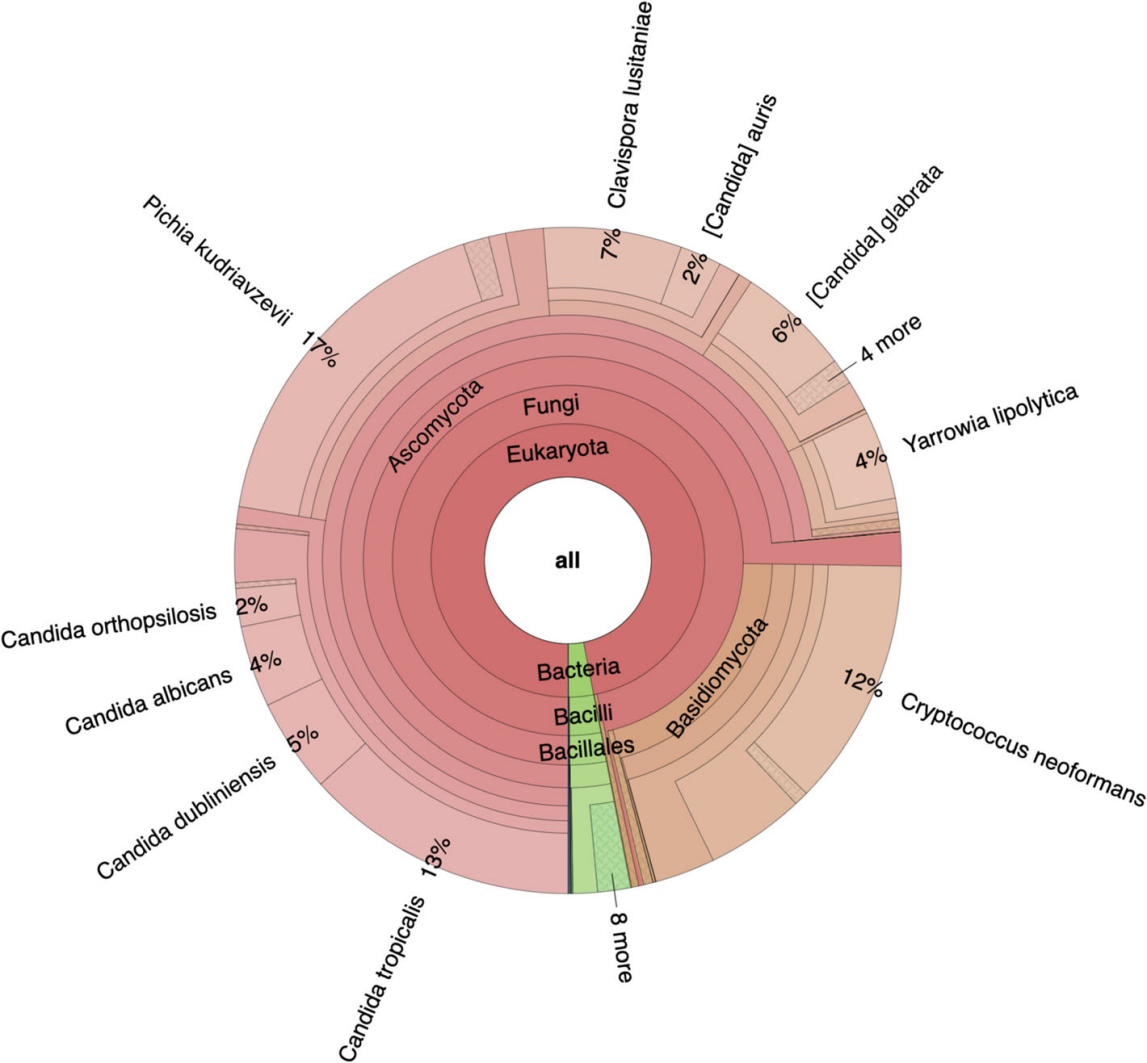
Snapshot of CCMetagen results for a spiked fungal community. This Krona graph shows the relative abundance of taxa at various taxonomic levels that are color-coded according to their taxonomic classification at lower-ranks—here, we see fungal taxa in shades of red, and bacterial taxa in shades of green. The Krona html file can be opened and interactively inspected in a web browser. Each circle represents a taxonomic level, where the user can click for a representation of the relative abundance at a given taxonomic rank. For a detailed list of taxa, refer to Additional file 5

We validated the CCMetagen pipeline with a fungal community previously generated in vitro by culturing, processing, and sequencing 15 fungal species ([34], Additional file 5). The analyses were performed using the NCBI nt collection as a reference. Our pipeline correctly retrieved 13 of the 15 fungal species sequenced, in addition to identifying a small component of other eukaryotic (0.4%) and bacterial (3%) RNA, which likely represent laboratory contaminants (Fig. 4, Additional file 5).

As this data set contains the same 15 fungal species as those simulated in silico, it is possible to tease apart classification errors from laboratory-related confounders such as contamination. Accordingly, we were able to retrieve all 15 species when using the in silico data set, suggesting that the two false negatives (*Schizosaccharomyces pombe* and *Debaryomyces hansenii*) were missing due to laboratory-related issues, such as RNA extraction biases, gene [under] expression, and imprecise cell counts. We also identified seven times more false positives in the seeded fungal metatranscriptome (44 species, including bacteria, while the simulated data yielded only 6). These additional 38 species were present at low abundance and possibly represent reagent and laboratory contaminants [35, 36] as they were not identified in the analysis of the equivalent simulated metatranscriptome.

### Biological data set 2: Australian birds

**Fig. 5:**
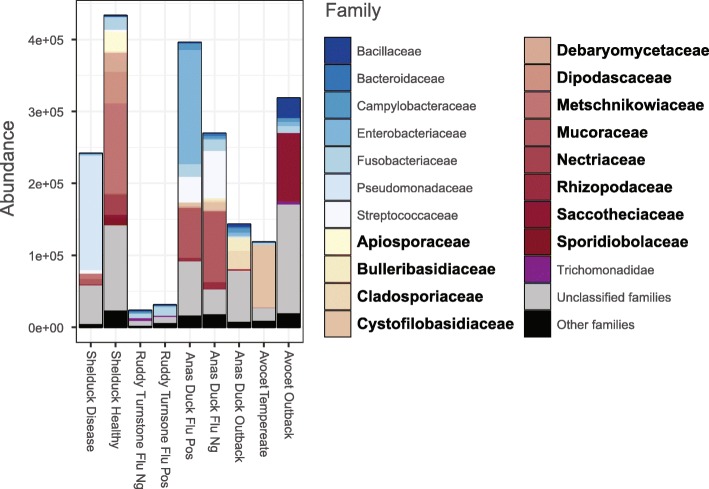
Microbial families in the microbiome of wild birds. The 20 most abundant families are shown, with fungal families indicated in bold. For a full list of taxa, refer to Additional file 6. A tutorial and R scripts to reproduce these analyses are available on the CCMetagen website

We used the CCMetagen pipeline to characterize the gut microbiome represented in 9 metatranscriptome libraries from wild birds sampled at various sites across Australia [37, 38]. These samples were collected as part of a long-term avian influenza study and were stored in Viral Transport Medium (brain-heart infusion broth containing 2 × 10^6^ IU/l penicillin, 0.2 mg/ml 383 streptomycin, 0.5 mg/ml gentamicin, 500 U/ml amphotericin B, Sigma), possibly simplifying microbiome composition and abundance, but not necessarily eliminating microbial genetic material. Indeed, fungal and bacterial transcripts were observed in all libraries (Additional file 6). Eukaryotic microbes accounted for 60% of the family-level diversity of the bird microbiome samples (taxa unclassified at family-level were not taken into account). Notably, fungi represented 12 of the 20 most abundant microbial families (Fig. 5). Among the fungal transcripts with a species-level classification, those attributed to the basidiomycete *Cystofilobasidium macerans* (Tremellomycetes) were the most abundant and were present in all bird libraries. Transcripts from species of filamentous fungi (e.g., *Mucor*, *Cladosporium*, *Fusarium*) and yeasts (e.g., *Cryptococcus*, *Metschnikowia*) were common. The high diversity of fungi associated with birds is unsurprising, as birds are known to play an important role in the ecology and distribution of yeasts and fungal spores [39, 40]. Bird excrement is a natural niche for species of the opportunistic pathogen *Cryptococcus* [41, 42], and several studies have reported *Mucor*, *Cladosporium*, and *Cryptococcus* associated with birds [43–45]. Species of *Fusarium* and *Metschnikowia* are often associated with plants and may be transient microbes in the avian microbiome, following ingestion of plant materials containing spores or dormant yeast cells [40, 46]. Other microbial eukaryotes were also observed, including the trichomonad *Simplicimonas* and the Apicomplexan *Eimeria*. Archaeal and viral transcripts were also detected. The methanogenic archaea *Methanobrevibacter woesei*, which was previously reported in chicken guts [47], was observed in two duck libraries. Influenza A virus was detected and confirmed with PCR-based methods [37]. The CCMetagen results were parsed with PhyloSeq for a graphical representation of the most abundant microbes, and the R script to reproduce Fig. 5 is available on the CCMetagen website [48].

## Discussion

The application of the ConClave sorting scheme to differentiate highly similar genetic sequences [16] represents an important step forward in metagenomic species profiling. We have applied this concept to develop a metagenome classification pipeline that is highly accurate yet fast enough to use the entire NCBI nucleotide collection as a reference, thereby facilitating the identification of microbial eukaryotes in metagenomic studies. The species-level identifications of bacteria and fungi obtained with the CCMetagen pipeline were from 3× to 1580× more precise than other metagenome classifiers (across all databases tested). CCMetagen is therefore a powerful tool for achieving accurate taxon identifications across a range of biological kingdoms in metagenome or metatranscriptome samples.

Scarce reference data pose a major challenge to study any microbial system that is less well-studied than the human gut. Some methods with reportedly high accuracy rely heavily on reference databases of complete or near-complete genomes. KrakenUniq, for example, showed relatively high precision and recall when using the RefSeq-bf database, which contained the complete genomes of all species in the test data set. However, when KrakenUniq was tested with an incomplete reference database (RefSeq-f-partial), the number of false positives increased, on average, from 51 to 221 species. This likely happens because it is relatively easy to identify a species that is present in the reference database, while it can be challenging to identify the closest match in the absence of a perfectly matching reference sequence. In the latter case, when reads are classified individually, multiple reference sequences can have identical levels of similarity, leading to a high number of false positives. This is an obvious problem when working with microbial eukaryotes, for which very few complete genomes are available.

One of the many advantages of metagenomics is that it enables the detection of both novel and rare microbes. Being able to distinguish between known and novel microorganisms in metagenomic data sets is a desirable feature possessed by surprisingly few metagenome classifiers. Some of these classifiers (e.g., MEGAN and Kraken) use the lowest common ancestor between all reference sequences that match the query sequence. The accuracy of these taxonomic classifiers tends to decrease as reference databases get populated with closely related taxa [14], and paradoxically, well-known taxa can be classified at higher taxonomic ranks than rare or novel ones. CCMetagen classifies taxa at the lowest common ancestor that reflects the genetic similarity between the query and the reference sequence. As rates of molecular evolution can vary substantially among genes and species, it is currently not feasible to set a universal sequence similarity threshold that works equally well for all organisms and genes. By default, CCMetagen uses similarity thresholds previously determined for fungi [49, 50]. Importantly, CCMetagen allows the user to easily set different similarity thresholds or disable the threshold-filtering step entirely. While this strategy also has limitations, it is a better alternative to the reference-dependent method of calculating LCAs, even when using the default thresholds for bacterial classifications (Fig. 3).

With CCMetagen, it is possible to confidently use metagenomics to identify microbial eukaryotes and prokaryotes in microbial communities. Our analyses of the gut microbiome of wild birds revealed an abundant and diverse community of micro-eukaryotes, representing 60% of the family-level diversity in the samples. We detected various species of *Mucor* and of basidiomycetes, including species of the opportunistic pathogen genus *Cryptococcus*. These and other non-ascomycetes fungi can be affected by mismatches in commonly used metabarcoding primers [51–53]. The fact that they were observed in high abundance indicates that metagenomics and metatranscriptomics are valuable for detecting these organisms in environmental samples. A recent analysis of 38 human gut microbiome samples using the CCMetagen pipeline and the NCBI nt database revealed only three fungal taxa (Saccharomycetaceae, Rhizopodaceae, and one unidentified family in the Dothideomycetes) in three samples [54], supporting the notion that the high diversity and abundance of fungi observed here is a feature of the avian microbiome rather than an artifact of the analysis. Importantly, CCMetagen can generate results in a format that resembles an operational taxonomic unit (OTU) table that can be imported into software designed for microbial community analyses, such as PhyloSeq [19], facilitating downstream ecological and statistical analyses of the microbiome.

## Conclusion

In summary, CCMetagen is a versatile pipeline implementing the ConClave sorting scheme (via KMA) to achieve more accurate taxonomic classifications than current analytic methods. The pipeline is fast enough to use the entire NCBI nt collection as the reference, facilitating the inclusion of understudied organisms, such as microbial eukaryotes, in metagenome surveys. CCMetagen produces ranked taxonomic results in user-friendly formats that are ready for publication (with Krona) or for downstream statistical analyses (with PhyloSeq). The pipeline is freely available as a web service and as a command line application. We expect that a range of novel ecological and evolutionary insights will be obtained as information about microbial eukaryotes in metagenomic studies becomes more accessible.

## Methods

### CCMetagen workflow and implementation

CCMetagen is a workflow implemented in Python 3 (Python ≥ 3.6). The analysis requires a reference database in which sequence headers contain taxonomic identifiers (taxids). Ready-to-use reference databases (NCBI nt and RefSeq) and instructions to create custom reference databases are provided in the CCMetagen website: https://github.com/vrmarcelino/CCMetagen [21]. Sequence reads, contigs, or long reads are first mapped to the reference database with KMA [16], which accepts single-end or paired-end, fastA, fastQ, and compressed (gzip) formats. CCMetagen is then used to process the KMA results via two main programs: *CCMetagen.py* and *CCMetagen_merge.py*. The first command takes as input the results of KMA and performs a customized quality control where the user can specify the minimum requirements to accept a match in terms of sequence depth, coverage, and ConClave scores. The pipeline will detect two (or more) closely related lineages if there are detectable SNP differences between the consensus alignments (between query sequences and templates). Supposing that Fig. 1b (Step 1) referred to two closely related species with different abundances, the pipeline would not detect them as separate taxa, as there are no detectable differences between them.

The *CCMetagen.py* program then processes taxonomic information using the ETE toolkit [55] and outputs a ranked taxonomic table—where taxon names for superkingdom, kingdom, phylum, class, order, family, genus, and species are attributed when known. Sequence similarity of the consensus alignment between query sequences and the template is calculated with KMA. *CCMetagen.py* applies a sequence similarity threshold to define the lowest taxonomic rank that can be attributed with confidence. The default thresholds are based on large-scale analyses of fungal sequences [49, 50] and can be changed or disabled (so that no similarity filtering is performed) using built-in options in *CCMetagen.py*. The program provides the option to convert abundance units to the commonly used reads per million (RPM), and to produce interactive graphs showing the relative abundance of taxa using Krona [17]. After processing individual samples with *CCMetagen.py*, the user can use *CCMetagen_merge.py* to produce a single spreadsheet containing the results of all samples in comma-separated values (CSV) format. This spreadsheet reassembles an operational taxonomic unit (OTU) table, helping to integrate the CCMetagen results with existing statistical software designed for microbiome analysis (e.g., PhyloSeq [19]). *CCMetagen_merge.py* provides the option to merge taxa at different taxon ranks and to include or exclude taxa. A step-by-step tutorial on the CCMetagen workflow is provided online (https://github.com/vrmarcelino/CCMetagen/tree/master/tutorial [48]), and a web server version of CCMetagen, which requires no command line knowledge from the user, is available at https://cge.cbs.dtu.dk/services/ccmetagen/ [23].

### Test data sets

A fungal metagenome and a metatranscriptome were simulated in silico to assess the performance of CCMetagen and other classification pipelines in identifying the fungal members of a microbial community (Additional file 7). Simulations were based on complete fungal genomes obtained from the NCBI RefSeq collection [56]. The metagenome contained 30 fungal species and was simulated with Grinder [57] using parameters to mimic the insert size and sequencing errors of an Illumina library (-md poly4 3e-3 3.3e-8 -insert_dist 500 normal 50 -fq 1 -ql 30 10). Coverage was set to vary between 0.001× and 10× for different species. The simulated metagenome contained 6,767,167 PE reads (6,695,384 PE reads after quality control, see Additional file 3).

The metatranscriptome contained 15 fungal species and was simulated for a subsample of 4000 genes (CDSs) from each fungal genome. Transcripts were simulated with Polyester [58], using the Illumina5 error model and gene expression following a normal distribution of average 3× (20% of genes up- and 20% downregulated). The simulated fungal metatranscriptome contained 9,009,121 PE reads (9,008,363 PE reads after quality control, see Additional file 3).

Additionally, 10 bacterial metagenomes simulated by Segata et al. [32], and compiled in McIntyre et al. [33], were used to assess the performance of the different classifiers in identifying prokaryotic communities with various levels of complexity. Each metagenome contained between 25 and 100 bacterial species [33].

### Reference databases

Reference databases were downloaded and indexed as described in Additional file 3. We used three reference databases: (i) “nt”—the NCBI nucleotide collection [20]; (ii) “RefSeq-bf,” containing curated genomes of fungi (all assembly levels) and bacteria (only complete) in the NCBI Reference Sequence Database [56]; and (iii) “RefSeq-f-partial,” which is a subset of RefSeq-bf, containing only part of the fungal species in our test data sets. The RefSeq-f-partial database was built to assess how the programs perform when reference databases are incomplete, for example, when dealing with species without reference genomes. Fifteen species were removed, resulting in a database that contained 15 of the 30 species in the fungal metagenome sample, and 7 of the 15 species in the metatranscriptome sample (species removed from this data set are listed in Additional file 8). The nt and RefSeq-bf databases indexed to function with KMA and CCMetagen are hosted in two sites, at 10.25910/5cc7cd40fca8e [59] (Australia) and http://www.cbs.dtu.dk/public/CGE/databases/CCMetagen/ [60] (Denmark).

### Benchmarking

Details about the quality control and data analyses are described in Additional file 3. Metagenome classifications using Kraken2 v.2.0.6-beta, KrakenUniq v.0.5.6, and Centrifuge v.1.0.3-beta were performed using default values. The performance of the classifiers was assessed in terms of precision, recall, F1 score, and CPU time. Precision was calculated with the formula:

$$ \mathrm{Precision}=\frac{\mathrm{True}\ \mathrm{Positives}}{\mathrm{True}\ \mathrm{Positives}+\mathrm{False}\ \mathrm{Positives}} $$

Recall was calculated with the formula:

$$ \mathrm{Recall}=\frac{\mathrm{True}\ \mathrm{Positives}}{\mathrm{True}\ \mathrm{Positives}+\mathrm{False}\ \mathrm{Negatives}} $$

F1 score, which is the harmonic average of the precision and recall, was calculated as:

$$ \mathrm{F}1=2\ \mathrm{x}\ \frac{\mathrm{Precision}\times \mathrm{Recall}}{\mathrm{Precision}+\mathrm{Recall}} $$

True positives reflect the number of taxa in the test data set that was retrieved by the analysis. Likewise, false positives refer to the number of taxa that were identified in the analysis but were not present in the test data set, while false negatives are taxa present in the test data set that were not detected by the analysis. The accuracy of abundance estimates was not benchmarked in this study. Precision and recall were multiplied by 100 to indicate percentages. Precision, recall, and F1 scores were calculated at the levels of species, genus, family, order, class, and phylum, following the hierarchy of the NCBI taxonomic database [18]. Only matches to organisms with valid taxids were included in the analyses. Valid but obsolete taxids (altered due to nomenclature changes) were updated accordingly using the ETE toolkit [55]. This strategy also minimizes nomenclature problems. For example, *Filobasidiella neoformans* is a life stage of *Cryptococcus neoformans*; they share a unique taxid (5207) regardless of the name attributed to the sequence in the reference database. The benchmarking scripts are available at https://github.com/vrmarcelino/CCMetagen/tree/master/BenchmarkingTools.

### CCMetagen applied to real data sets

We validated the CCMetagen pipeline using two biological data sets: one defined fungal community (biological data set 1) and one set of environmental samples (biological data set 2). The fungal community was constructed by culturing, pooling, and sequencing the same 15 fungal species used in the metatranscriptome simulated in silico (SRA BioProject number PRJNA521097) [34].

The biological data set 2 consisted of nine metatranscriptome libraries derived from gut samples from Australian wild birds (SRA BioProject number PRJNA472212) [37]. Quality control was performed as described in Marcelino et al. [38].

These samples were mapped to the NCBI nucleotide database using KMA with the options -1t1 -mem_mode -and -apm f, and then processed with CCMetagen using default values. The results were parsed with PhyloSeq to produce a graph with taxa abundances (Fig. 5). A tutorial explaining the full analyses of the bird microbiome, from quality control to graphical representation with PhyloSeq, is available at https://github.com/vrmarcelino/CCMetagen/tree/master/tutorial.

## Supplementary information

**Additional file 1: Figure S1.** Precision of the different methods, using three reference databases. **Figure S2:** Recall of fungal taxa from a metagenome and a metatranscriptome test dataset.

**Additional file 2: Table S1.** Precision, recall and F1 scores obtained for fungal communities.

**Additional file 3:** Supplementary Materials and Methods.

**Additional file 4: Table S2.** Precision, recall and F1 scores obtained with the CCMetagen analysis with assembled sequence reads.

**Additional file 5: Table S3.** Species and transcripts observed in the metatranscriptome of a mock fungal community (biological data set 1).

**Additional file 6: Table S4.** Species observed in the metatranscriptome of wild birds (biological data set 2) and their abundance.

**Additional file 7: Table S5.** Genome sequences and species used to simulate fungal communities.

**Additional file 8: Table S6.** Fungal genome sequences removed from the RefSeq fungi database (RefSeq-f-partial) to mimic the effects of classifying species without reference genomes.

**Additional file 9:** Review history.

## Data Availability

CCMetagen source code is freely available from https://github.com/vrmarcelino/CCMetagen [21] (licensed under GNU General Public License v3.0) or via the Python Package Index PyPi [22]. The CCMetagen web server is available at https://cge.cbs.dtu.dk/services/ccmetagen/ [23]. The CCMetagen version used in this study is available in GitHub and Zenodo [61]. The simulated fungal metagenome and metatranscriptome sequence are available at 10.25910/5cc7cd40fca8e [59] (simulated_datasets.zip). The biological data is available on GenBank (SRA BioProject numbers PRJNA521097 [34] and PRJNA472212 [37]). The nt and RefSeq-bf databases indexed to function with KMA and CCMetagen are hosted in two sites, at 10.25910/5cc7cd40fca8e [59] (Australia) and http://www.cbs.dtu.dk/public/CGE/databases/CCMetagen/ [60] (Denmark). Scripts used to benchmark the software are available at https://github.com/vrmarcelino/CCMetagen/tree/master/benchmarking.

## References

[1] Caporaso JG, Lauber CL, Walters WA, Berg-Lyons D, Lozupone CA, Turnbaugh PJ (2011). Global patterns of 16S rRNA diversity at a depth of millions of sequences per sample. Proc Natl Acad Sci U S A.

[2] Taberlet P, Coissac E, Pompanon F, Brochmann C, Willerslev E (2012). Towards next-generation biodiversity assessment using DNA metabarcoding. Mol Ecol.

[3] Bik HM, Porazinska DL, Creer S, Caporaso JG, Knight R, Thomas WK (2012). Sequencing our way towards understanding global eukaryotic biodiversity. Trends Ecol Evol.

[4] Norman JM, Handley SA, Virgin HW (2014). Kingdom-agnostic metagenomics and the importance of complete characterization of enteric microbial communities. Gastroenterology..

[5] Marcelino VR, Verbruggen H (2016). Multi-marker metabarcoding of coral skeletons reveals a rich microbiome and diverse evolutionary origins of endolithic algae. Sci Rep.

[6] Piganeau G, Eyre-Walker A, Jancek S, Grimsley N, Moreau H (2011). How and why DNA barcodes underestimate the diversity of microbial eukaryotes. PLoS One.

[7] Breitwieser FP, Lu J, Salzberg SL (2019). A review of methods and databases for metagenomic classification and assembly. Brief Bioinform.

[8] Scholz M, Ward DV, Pasolli E, Tolio T, Zolfo M, Asnicar F (2016). Strain-level microbial epidemiology and population genomics from shotgun metagenomics. Nat Methods.

[9] Truong DT, Franzosa EA, Tickle TL, Scholz M, Weingart G, Pasolli E (2015). MetaPhlAn2 for enhanced metagenomic taxonomic profiling. Nat Methods.

[10] Sczyrba A, Hofmann P, Belmann P, Koslicki D, Janssen S, Droge J (2017). Critical assessment of metagenome interpretation - a benchmark of metagenomics software. Nat Methods.

[11] Nilsson RH, Anslan S, Bahram M, Wurzbacher C, Baldrian P, Tedersoo L (2019). Mycobiome diversity: high-throughput sequencing and identification of fungi. Nat Rev Microbiol.

[12] Huson DH, Auch AF, Qi J, Schuster SC (2007). MEGAN analysis of metagenomic data. Genome Res.

[13] Wood DE, Salzberg SL (2016). Kraken: ultrafast metagenomic sequence classification using exact alignments. Genome Biol.

[14] Nasko DJ, Koren S, Phillippy AM, Treangen TJ (2018). RefSeq database growth influences the accuracy of k-mer-based lowest common ancestor species identification. Genome Biol.

[15] Hawksworth DL, Lucking R (2017). Fungal diversity revisited: 2.2 to 3.8 million species. Microbiol. Spectr..

[16] Clausen P, Aarestrup FM, Lund O (2018). Rapid and precise alignment of raw reads against redundant databases with KMA. BMC Bioinformatics.

[17] Ondov BD, Bergman NH, Phillippy AM (2011). Interactive metagenomic visualization in a web browser. BMC Bioinformatics..

[18] Federhen S (2012). The NCBI Taxonomy database. Nucleic Acids Res.

[19] McMurdie PJ, Holmes S (2013). phyloseq: an R package for reproducible interactive analysis and graphics of microbiome census data. PLoS One.

[20] Mizrachi I. GenBank: the nucleotide sequence database. The NCBI handbook [Internet], updated (2007).

[21] Marcelino VR, Clausen PTLC, Buchman J, Wille M, Iredell JR, Meyer W, et al. CCMetagen GitHub repository. https://github.com/vrmarcelino/CCMetagen (2019).

[22] Buchman J, Marcelino VR, Clausen PT, Wille M, Iredell JR, Meyer W, et al. CCMetagen Python Package Index. https://pypi.org/project/CCMetagen/ (2020).

[23] Clausen PTLC, Marcelino VR, Buchman J, Wille M, Iredell JR, Meyer W, et al. CCMetagen webserver. https://cge.cbs.dtu.dk/services/ccmetagen/ (2019).

[24] Kim D, Song L, Breitwieser FP, Salzberg SL (2016). Centrifuge: rapid and sensitive classification of metagenomic sequences. Genome Res.

[25] Wood DE, Lu J, Langmead B (2019). Improved metagenomic analysis with Kraken 2. Genome Biol.

[26] Breitwieser FP, Baker DN, Salzberg SL (2018). KrakenUniq: confident and fast metagenomics classification using unique k-mer counts. Genome Biol.

[27] Altschul SF, Gish W, Miller W, Myers EW, Lipman DJ (1990). Basic local alignment search tool. J Mol Biol.

[28] Buchfink B, Xie C, Huson DH (2015). Fast and sensitive protein alignment using DIAMOND. Nat Methods.

[29] Ounit R, Wanamaker S, Close TJ, Lonardi S (2015). CLARK: fast and accurate classification of metagenomic and genomic sequences using discriminative k-mers. BMC Genomics.

[30] Freitas TA, Li PE, Scholz MB, Chain PS (2015). Accurate read-based metagenome characterization using a hierarchical suite of unique signatures. Nucleic Acids Res.

[31] Darling AE, Jospin G, Lowe E, Matsen FA, Bik HM, Eisen JA (2014). PhyloSift: phylogenetic analysis of genomes and metagenomes. PeerJ..

[32] Segata N, Waldron L, Ballarini A, Narasimhan V, Jousson O, Huttenhower C (2012). Metagenomic microbial community profiling using unique clade-specific marker genes. Nat Methods.

[33] McIntyre ABR, Ounit R, Afshinnekoo E, Prill RJ, Henaff E, Alexander N (2017). Comprehensive benchmarking and ensemble approaches for metagenomic classifiers. Genome Biol.

[34] Marcelino VR, Irinyi L, Eden J-S, Meyer W, Holmes EC, Sorrell TC (2019). Metatranscriptomics as a tool to identify fungal species and subspecies in mixed communities – a proof of concept under laboratory conditions. IMA Fungus.

[35] Salter SJ, Cox MJ, Turek EM, Calus ST, Cookson WO, Moffatt MF (2014). Reagent and laboratory contamination can critically impact sequence-based microbiome analyses. BMC Biol.

[36] Strong MJ, Xu G, Morici L, Splinter Bon-Durant S, Baddoo M, Lin Z (2014). Microbial contamination in next generation sequencing: implications for sequence-based analysis of clinical samples. PLoS Path.

[37] Wille M, Eden JS, Shi M, Klaassen M, Hurt AC, Holmes EC (2018). Virus-virus interactions and host ecology are associated with RNA virome structure in wild birds. Mol Ecol.

[38] Marcelino VR, Wille M, Hurt AC, Gonzalez-Acuna D, Klaassen M, Schlub TE (2019). Meta-transcriptomics reveals a diverse antibiotic resistance gene pool in avian microbiomes. BMC Biol.

[39] Moschetti G, Alfonzo A, Francesca N, Buzzini P, Lachance M-A, Yurkov A (2017). Yeasts in birds. Yeasts in natural ecosystems: diversity.

[40] Evans RN, Prusso DC (1969). Spore dispersal by birds. Mycologia..

[41] Nielsen K, De Obaldia AL, Heitman J (2007). *Cryptococcus neoformans* mates on pigeon guano: implications for the realized ecological niche and globalization. Eukaryot Cell.

[42] Cafarchia C, Romito D, Iatta R, Camarda A, Montagna MT, Otranto D (2006). Role of birds of prey as carriers and spreaders of *Cryptococcus neoformans* and other zoonotic yeasts. Med Mycol.

[43] Hubalek Z (1978). Coincidence of fungal species associated with birds. Ecology..

[44] Rosario I, Hermoso de Mendoza M, Deniz S, Soro G, Alamo I, Acosta B (2005). Isolation of *Cryptococcus* species including *C. neoformans* from cloaca of pigeons. Mycoses..

[45] Hargreaves J, Brickle P, van West P (2018). The fungal ecology of seabird nesting sites in the Falkland Islands indicates a niche for mycoparasites. Fungal Ecol.

[46] Correia M, Heleno R, da Silva LP, Costa JM, Rodriguez-Echeverria S (2019). First evidence for the joint dispersal of mycorrhizal fungi and plant diaspores by birds. New Phytol.

[47] Saengkerdsub S, Anderson RC, Wilkinson HH, Kim WK, Nisbet DJ, Ricke SC (2007). Identification and quantification of methanogenic Archaea in adult chicken ceca. Appl Environ Microbiol.

[48] Marcelino VR, Clausen PTLC, Buchman J, Wille M, Iredell JR, Meyer W, et al. CCMetagen tutorial. https://github.com/vrmarcelino/CCMetagen/tree/master/tutorial (2019).

[49] Vu D, Groenewald M, de Vries M, Gehrmann T, Stielow B, Eberhardt U (2019). Large-scale generation and analysis of filamentous fungal DNA barcodes boosts coverage for kingdom fungi and reveals thresholds for fungal species and higher taxon delimitation. Stud Mycol.

[50] Vu D, Groenewald M, Szoke S, Cardinali G, Eberhardt U, Stielow B (2016). DNA barcoding analysis of more than 9 000 yeast isolates contributes to quantitative thresholds for yeast species and genera delimitation. Stud Mycol.

[51] Tedersoo L, Lindahl B (2016). Fungal identification biases in microbiome projects. Environ Microbiol Rep.

[52] Ihrmark K, Bodeker IT, Cruz-Martinez K, Friberg H, Kubartova A, Schenck J (2012). New primers to amplify the fungal ITS2 region--evaluation by 454-sequencing of artificial and natural communities. FEMS Microbiol Ecol.

[53] Bellemain E, Carlsen T, Brochmann C, Coissac E, Taberlet P, Kauserud H (2010). ITS as an environmental DNA barcode for fungi: an *in silico* approach reveals potential PCR biases. BMC Microbiol.

[54] Marcelino VR, Holmes EC, Sorrell TC (2020). The use of taxon-specific reference databases compromises metagenomic classification. BMC Genomics.

[55] Huerta-Cepas J, Serra F, Bork P (2016). ETE 3: reconstruction, analysis, and visualization of phylogenomic data. Mol Biol Evol.

[56] Pruitt KD, Tatusova T, Maglott DR (2007). NCBI reference sequences (RefSeq): a curated non-redundant sequence database of genomes, transcripts and proteins. Nucleic Acids Res.

[57] Angly FE, Willner D, Rohwer F, Hugenholtz P, Tyson GW (2012). Grinder: a versatile amplicon and shotgun sequence simulator. Nucleic Acids Res.

[58] Frazee AC, Jaffe AE, Langmead B, Leek JT (2015). Polyester: simulating RNA-seq datasets with differential transcript expression. Bioinformatics..

[59] Marcelino VR, Clausen PTLC, Buchman J, Wille M, Iredell JR, Meyer W, et al. Indexed reference databases for KMA and CCMetagen. 10.25910/5cc7cd40fca8e (2019).

[60] Clausen PTLC, Marcelino VR, Buchman J, Wille M, Iredell JR, Meyer W, et al. Indexed reference databases for KMA and CCMetagen - mirror. http://www.cbs.dtu.dk/public/CGE/databases/CCMetagen/ (2019).

[61] Marcelino VR, Clausen PTLC, Buchman J, Wille M, Iredell JR, Meyer W, et al. Zenodo repository of CCMetagen v 1.0.0. 10.5281/zenodo.3668497 (2020).

