# Supplementary material for: CCMetagen: comprehensive and accurate identification of eukaryotes and prokaryotes in metagenomic data
Source: Genome Biol. 2020 Apr 28;21:103. doi: 10.1186/s13059-020-02014-2 (PMC7189439; doi:10.1186/s13059-020-02014-2)
Supplement: Supplementary file 1 — Additional file 1: Figure S1. Precision of the different methods, using three reference databases. Figure S2: Recall of fungal taxa from a metagenome and a metatranscriptome test dataset. [file 13059_2020_2014_MOESM1_ESM.pdf]

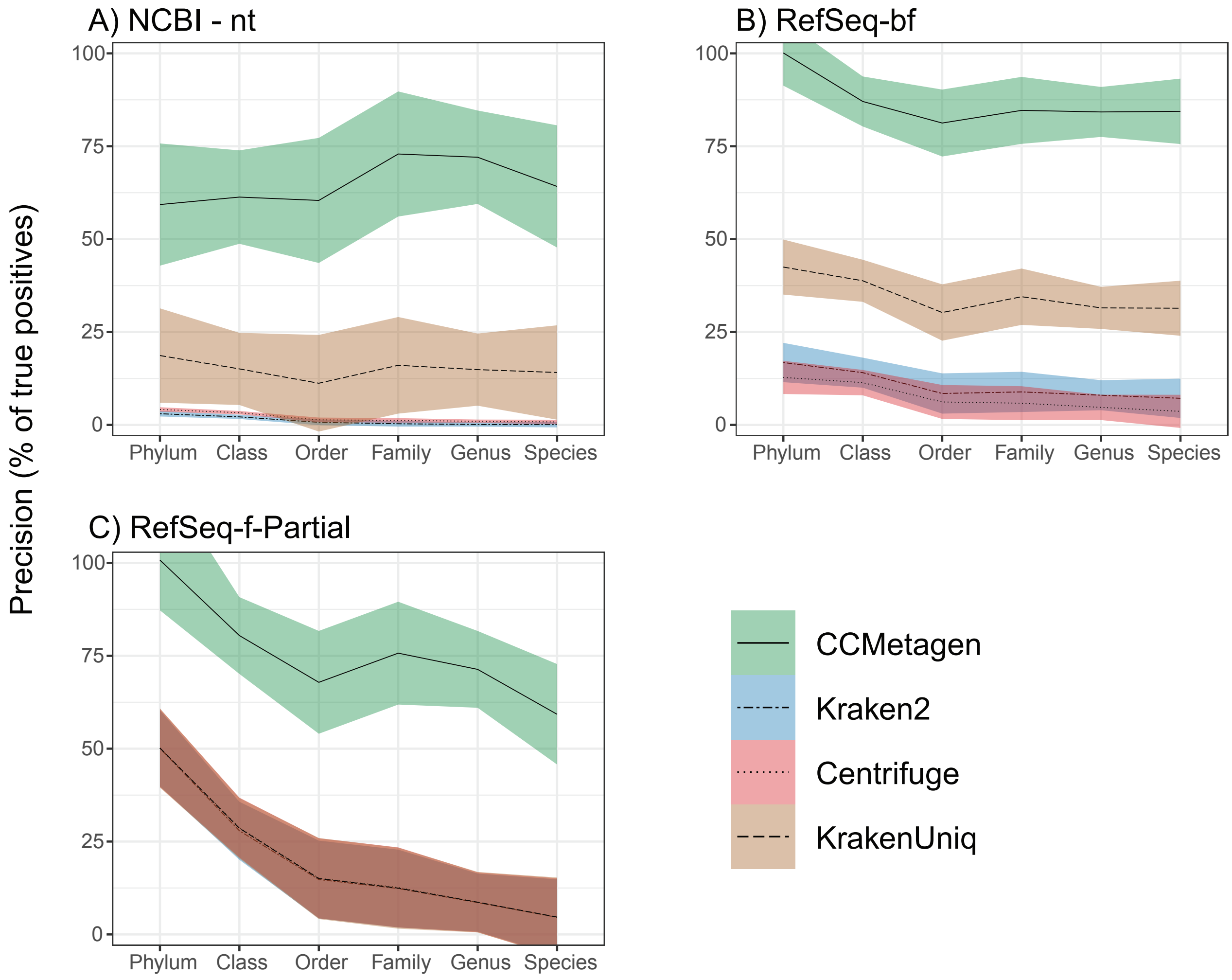

Supplemental Figure S1. Precision of the different methods, using three reference databases. (A) Results using the whole NCBI's nt collection as a reference database. (B) Results using the RefSeq (bacteria fungi) database, containing all bacterial and fungal genomes available.
(C) Partial RefSeq (fungi - partial) database, which mimics the effects of dealing with species without representatives in reference datasets. Kraken2, Centrifuge and KrakenUniq have overlapping results when using the Partial RefSeq database.
Shaded areas indicate 75% confidence intervals.

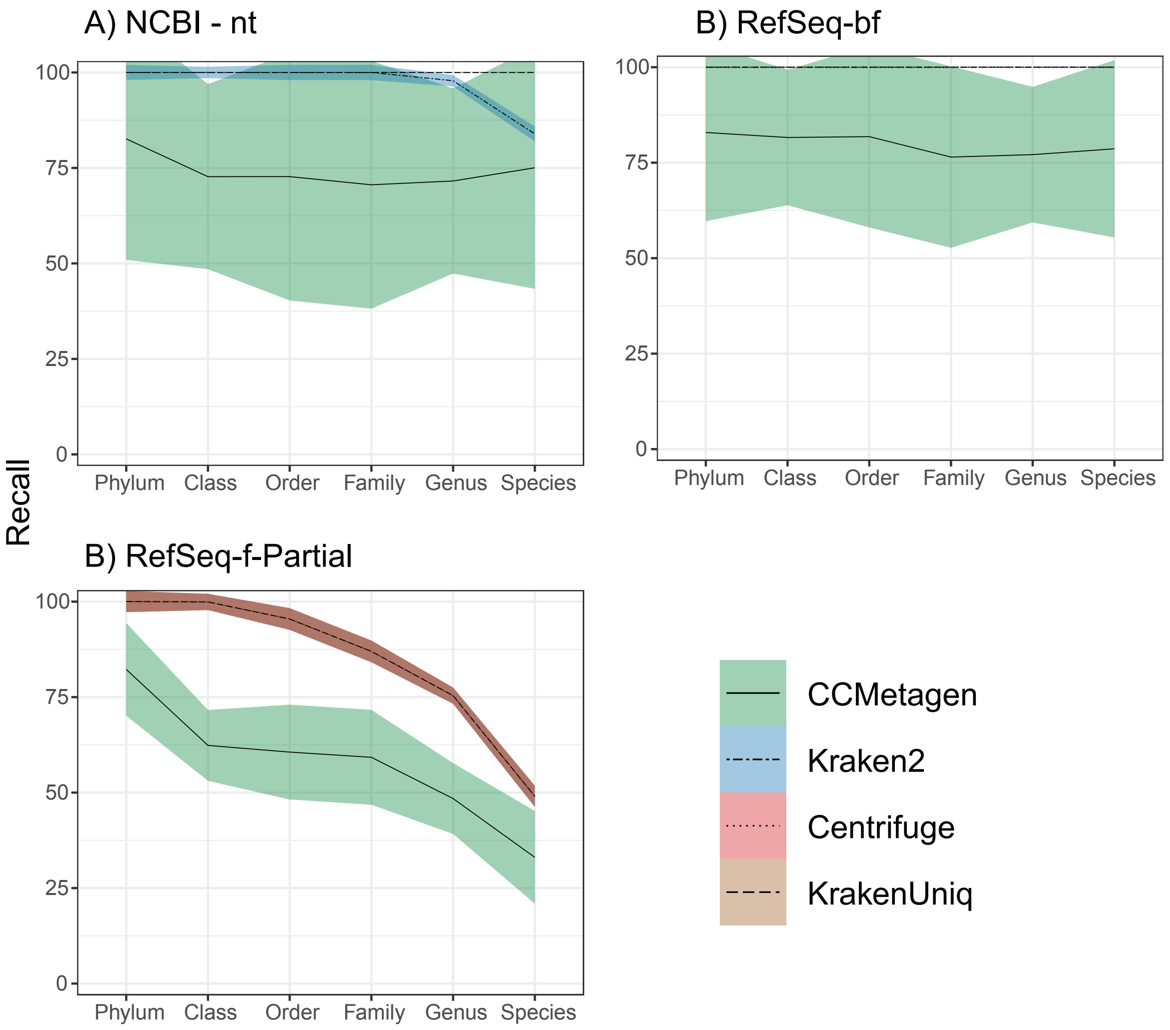

Supplemental Figure S2. Recall (% of taxa identified) of fungal taxa from a metagenome and a metatranscriptome test dataset. Note that the recall of Centrifuge, KrakenUniq and Kraken2 (up to genus level) is 100% (one straight line on top of graphs A and B).
The brown shaded area in C represents the overlapping results of Kraken2, Centrifuge and KrakenUniq. Shaded areas indicate 75% confidence intervals.
