# Supplementary material for: CCMetagen: comprehensive and accurate identification of eukaryotes and prokaryotes in metagenomic data
Source: Genome Biol. 2020 Apr 28;21:103. doi: 10.1186/s13059-020-02014-2 (PMC7189439; doi:10.1186/s13059-020-02014-2)
Supplement: Supplementary file 9 — Additional file 9: Review history. [file 13059_2020_2014_MOESM9_ESM.docx]

Review History

**First round of review**

**Reviewer 1**

**Were you able to assess all statistics in the manuscript, including the appropriateness of statistical tests used?**

Yes, and I have assessed the statistics in my report.

**Were you able to directly test the methods?**

Yes

**Comments to author:**

The manuscript by Marcelino et al. describes a new tool for metagenomic taxonomic classification - CCMetagen - and shows that the method works better than existing commonly used tools on a few reasonable chosen datasets - real as well as simulated. Overall, the methodology is sound, the manuscript is well written and the conclusions well supported. I have a few minor questions and ideas that might improve the manuscript. However, I also have a concern that there might not be that much novelty to it. Essentially, the authors use an existing mapping software and classification algorithm (KMA; previously published in BMC Bioinformatics) combined with a published database curated by NCBI to generate taxonomic output. While I see the value of their pipeline, I wonder if the work reaches the threshold to appeal to the wide readership of Genome Biology.

Other than that, this is a great manuscript and a useful tool, and if I only were to consider soundness of the paper, I would recommend publication after some minor tweaks (see below).

Specific comments

-	A general question on the KMA/CC algorithm: How are cases where you have mapping across two entire sequences, but potentially with different abundances, handled? (As opposed to uneven coverage as shown in Figure 1.)

-	Line 66-67: Actually, this is not really affecting the analysis per se negatively. Rather, the previous relative success of these other methods was based on a limited knowledge of genome data... Perhaps this should be rephrased?

-	Line 95-96: How is the similarity level that reflect appropriate classification determined?

-	Figure 2: The data shown in Figure 2 is also a pretty good argument for why NT should not really be used as a database for taxonomic classification. I think this could be stressed, because it is quite clear from the data.

-	Line 162-163: What effect does the prefix-indexing have on the sensitivity? I think a number on that would be quite interesting to a user deciding which parameters to choose.

-	Table 1 and line 181-184: How many reads where in each of the simulated metagenomes? That's quite important to interpret these running times.

-	Figure 3: It would be interesting to see these results compared to a specialized database, such as human gut data mapped to a database of human microbiome project genomes. Would that be hard to add?

-	Line 217: Couldn't these 38 species also be misclassifications?

-	Lien 226-227: I am no expert on the gut microbiomes of birds, but does it make sense that fungi are so abundant in the bird gut microbiome? It would make no sense in the human microbiome, but perhaps it does in birds? Could the authors comment on that?

-	Line 252-253: How does this pipeline compare to an approach where marker genes are extracted from the metagenomes, such as mOTU or metaxa2? It would be interesting to see such a comparison, even though I realize that it might be a fairly big addition to the manuscript.

-	Line 326-329: How were these databases retrieved from NCBI?

**Reviewer 2**

**Were you able to assess all statistics in the manuscript, including the appropriateness of statistical tests used?**

Yes, and I have assessed the statistics in my report.

**Were you able to directly test the methods?**

Yes

**Comments to author:**

Overall, I think CCmetagen is an important contribution to the field of metagenomic annotation that addresses an important issue of how to annotate across redundant databases.

The paper summarizes how KMA and the ConCave method works on metagenomic reads. However, there as almost no actual description of how CCmetagen works. Lines 95-96 and an image in Figure two are as specific a description as I could find but they do not describe the method. The method should be clearly described to the reader.

The benchmarking section 340-366 provides users the standard definitions of common metrics like precision, recall, and F1. But the way that a classification success or failure is being defined needs more explanation. Some of these programs return multiple classifications and there are different levels of classification. The recall scores are 100% for some classifiers like Kraken were explained by how many taxa were identified. This makes me wonder if PRC scores were calculated on the total annotations and not on a per read basis.

It would be very valuable to run the classifier comparison on long read data or assembled data. The is mentioned in in line 90 but the analysis was not done.

Suggestions:

The installation of command-line CCmetagen was not too complicated, but it could easily be added bioconda to make it easier for users.

The online server application is beneficial for evaluating the classifier and for small data sets. It would be helpful to mention that gzipped files are supported and the newer NT database with environmental sequences removed should be provided for users.

It would be helpful to generate a DOI for the version of software being evaluated in Zenodo.

**Authors Response**

**Point-by-point responses to the reviewers’ comments:**

Reviewer reports:

Reviewer #1: The manuscript by Marcelino et al. describes a new tool for metagenomic taxonomic classification - CCMetagen - and shows that the method works better than existing commonly used tools on a few reasonable chosen datasets - real as well as simulated. Overall, the methodology is sound, the manuscript is well written and the conclusions well supported. I have a few minor questions and ideas that might improve the manuscript. However, I also have a concern that there might not be that much novelty to it. Essentially, the authors use an existing mapping software and classification algorithm (KMA; previously published in BMC Bioinformatics) combined with a published database curated by NCBI to generate taxonomic output. While I see the value of their pipeline, I wonder if the work reaches the threshold to appeal to the wide readership of Genome Biology.

*>> Response: We thank the reviewer for their feedback and have addressed their questions and suggestions. We now provide a more comprehensive description of the CCMetagen pipeline that better highlights the novelty of this study.*

*>> This is the first time that the ConClave sorting scheme has been implemented for taxonomic profiling, and our pipeline enables the straightforward use of this novel method in routine metagenome analysis – featuring command line and webserver versions - that we believe will appeal to the readership of Genome Biology. Many microbiome pipelines were built upon existing tools and have proven to be very popular (e.g. MEGAN, Genome Research 2007, >2000 citations, based on BLAST). Additionally, to our knowledge, this is the first study to benchmark metagenome classifiers with complex eukaryotic communities, and provides simulated fungal metagenome and metatranscriptome datasets, which are valuable resources for a broad scientific community.*

Other than that, this is a great manuscript and a useful tool, and if I only were to consider soundness of the paper, I would recommend publication after some minor tweaks (see below).

Specific comments

- A general question on the KMA/CC algorithm: How are cases where you have mapping across two entire sequences, but potentially with different abundances, handled? (As opposed to uneven coverage as shown in Figure 1.)

*>> Response: This will depend on how similar the two sequences are and their abundance. If there are detectable SNP differences between the two taxa, the pipeline will detect them as separate taxa (if they pass the adjustable thresholds for score, depth and coverage). We have now included this explanation in the text:*

*“The pipeline will detect two (or more) closely related lineages if there are detectable SNP differences between the consensus alignments (between query sequences and templates). Supposing that Figure 1B – Step 1 referred to two closely-related species with different abundances, the pipeline would not detect them as separate taxa, as there are no detectable SNP differences between them.*

- Line 66-67: Actually, this is not really affecting the analysis per se negatively. Rather, the previous relative success of these other methods was based on a limited knowledge of genome data... Perhaps this should be rephrased?

*>> Response: We have deleted this confusing section of the sentence.*

- Line 95-96: How is the similarity level that reflect appropriate classification determined?

*>> Response: This is now provided in the ‘CCMetagen workflow and implementation’ subsection of the Methods. In short, sequence similarity is based on the consensus alignment between query sequences and the template, and is calculated with KMA. The default similarity levels used by CCMetagen are based on -published large-scale analyses of fungal sequences, and can be changed or disabled (so no similarity filtering is performed) using built-in options.*

- Figure 2: The data shown in Figure 2 is also a pretty good argument for why NT should not really be used as a database for taxonomic classification. I think this could be stressed, because it is quite clear from the data.

*>> Response: In our tests, the RefSeq database outperforms the NCBI nt, but we believe that is because RefSeq contains a smaller dataset of complete genomes. We opted to avoid discussing the choice of databases as this is a delicate issue that goes beyond the choice of the pipeline (i.e. CCMetagen can be used with any database).*

- Line 162-163: What effect does the prefix-indexing have on the sensitivity? I think a number on that would be quite interesting to a user deciding which parameters to choose.

*>> Response: We have now stated in the manuscript that choosing a longer prefix will result in gaps in the database which in turn will result in lower precision and recall. With a prefix of two this is relatively limited.*

- Table 1 and line 181-184: How many reads where in each of the simulated metagenomes? That's quite important to interpret these running times.

*>> Response: The number of reads is now given in the table caption and the methods section.*

- Figure 3: It would be interesting to see these results compared to a specialized database, such as human gut data mapped to a database of human microbiome project genomes. Would that be hard to add?

*>> Response: Unfortunately, this would require modifications in the sequence identifiers and indexing of these specialized reference databases for all programs tested. We agree with the reviewer that it would be valuable to compare these reference databases, but feel that this would be more appropriate as a separate publication.*

- Line 217: Couldn't these 38 species also be misclassifications?

*>> Response: While this possibility cannot be entirely excluded, we now clarify that these 38 species are more likely to be laboratory contaminants because we did not identify them in the analysis of a simulated metatranscriptome containing the same fungal species.*

- Lien 226-227: I am no expert on the gut microbiomes of birds, but does it make sense that fungi are so abundant in the bird gut microbiome? It would make no sense in the human microbiome, but perhaps it does in birds? Could the authors comment on that?

*>> Response: We have now made in clear in the manuscript that sample treatment may have influenced the microbiome composition (Lines 255–260), and that fungi are well-known inhabitants of the avian microbiome.*

*>> In addition, we have rephrased the section subheading and the text to avoid implying that our results are a representation of the bird microbiome, as our samples were collected for an avian influenza study and were stored in Viral Transport Media (which contains antibiotics and antifungals), likely altering the microbiome composition. Rather, we use these samples to show potential applications of our software and agree that they may not represent the Australian bird microbiome.*

*>> We also note that the abundant fungal taxa observed here were unsurprising, as most have been observed in association with birds before, and we now provide references to guide the interested reader (Lines 267¬ – 274).*

*>> Finally, in the discussion (Lines 333¬–338), we cite a recent study where we used CCMetagen and the NCBI nt collection to classify human metagenomes (Marcelino et al accepted, enclosed in this submission). In this study, we found fungi in only 3 samples and at very low abundances. This supports our contention that the high diversity and abundance of fungi observed here is a feature of the avian microbiome rather than an artefact of the analysis.*

- Line 252-253: How does this pipeline compare to an approach where marker genes are extracted from the metagenomes, such as mOTU or metaxa2? It would be interesting to see such a comparison, even though I realize that it might be a fairly big addition to the manuscript.

*>> Response: It is possible that the reference database used has a larger impact than the pipeline itself. Biases in the reference database will distort all approaches and we agree that a marker gene extraction might be helpful. However, this would be a very big addition to this study, beyond the scope of what would be reasonable to fit in this manuscript.*

*>> CCMetagen can already be used with the Unite ITS database (options -r UNITE), expanding it for easy use with other marker genes and benchmarking it with other software is in our plans for the future.*

- Line 326-329: How were these databases retrieved from NCBI?

*>> Response: The exact commands and links used to download the reference databases are provided in the supplementary materials (Additional file 3). We now refer to this file in the first sentence of this paragraph.*

Reviewer #2: Overall, I think CCmetagen is an important contribution to the field of metagenomic annotation that addresses an important issue of how to annotate across redundant databases.

The paper summarizes how KMA and the ConCave method works on metagenomic reads. However, there as almost no actual description of how CCmetagen works. Lines 95-96 and an image in Figure two are as specific a description as I could find but they do not describe the method. The method should be clearly described to the reader.

*>> Response: This is a good point. Therefore, we have now added a subsection detailing the CCMetagen implementation in the methods.*

The benchmarking section 340-366 provides users the standard definitions of common metrics like precision, recall, and F1. But the way that a classification success or failure is being defined needs more explanation. Some of these programs return multiple classifications and there are different levels of classification. The recall scores are 100% for some classifiers like Kraken were explained by how many taxa were identified. This makes me wonder if PRC scores were calculated on the total annotations and not on a per read basis.

*>> Response: We have expanded the Methods section to indicate that these metrics were based on the number of taxa observed rather than on a per read basis (Lines 446¬–450). The accuracy of the taxonomic predictions is, in our opinion, a clearer representation of the programs’ accuracy: an accuracy is 50%, for example, indicates that half of the taxa identified are false positives. We agree with the view that it would be valuable to benchmark the accuracy of the programs in estimating species abundance (where a read basis benchmarking would be more reasonable), but that would require an entire new set of analyses that go beyond the scope of our software manuscript. Kraken for example requires a second program (Bracken - Lu et al 2017) to estimate taxa abundances. We now explicitly state in the Methods section that “The accuracy of abundance estimates was not benchmarked in this study.”*

It would be very valuable to run the classifier comparison on long read data or assembled data. The is mentioned in line 90 but the analysis was not done.

*>> Response: We have included a CCMetagen analysis of two assembled data sets (the metagenome and metatranscriptome generated in this study). The results are incorporated in the Results section and in the additional files (Additional file 3 and Additional file1: Table S6). However, we have opted not to include the comparison with other programs, as Centrifuge do not scale well with long read data and Kraken and KrakenUniq were not designed to work with long read data, therefore a re-indexing would be required of those databases in order to give a reasonable comparison. Hence, we believe that these comparisons will only add confusion.*

Suggestions:

The installation of command-line CCmetagen was not too complicated, but it could easily be added bioconda to make it easier for users.

*>> Response: We added CCMetagen to the PyPi repository. It can be installed with pip, e.g. pip install ccmetagen.*

The online server application is beneficial for evaluating the classifier and for small data sets. It would be helpful to mention that gzipped files are supported and the newer NT database with environmental sequences removed should be provided for users.

*>> Response: Done.*

It would be helpful to generate a DOI for the version of software being evaluated in Zenodo.

*>> Response: Thanks for this suggestion. The software version used in this paper is now available at Zenodo (010.5281/zenodo.3668497) and github. This information is now in the ‘availability of data and materials’ section.*

**Second round of review**

**Reviewer 1**

I think that the authors have addressed my earlier concerns with the manuscript sufficiently well. Particularly, the extended methods section on how the tool operates significantly increased the quality of the paper.

If the editor is convinced that the manuscript is suitable for Genome Biology, is support its publication in the present form.
